# *Arabidopsis thaliana* responds to colonisation of *Piriformospora indica* by secretion of symbiosis-specific proteins

**DOI:** 10.1371/journal.pone.0209658

**Published:** 2018-12-27

**Authors:** Johannes Thürich, Doreen Meichsner, Alexandra C. U. Furch, Jeannette Pfalz, Thomas Krüger, Olaf Kniemeyer, Axel Brakhage, Ralf Oelmüller

**Affiliations:** 1 Plant Physiology, Matthias-Schleiden-Institute for Genetics, Bioinformatics and Molecular Botany, Faculty of Biological Science, Friedrich-Schiller-University Jena, Jena, Germany; 2 Molecular and Applied Microbiology, Leibniz Institute for Natural Product Research and Infection Biology Hans Knöll Institute, Jena, Germany; 3 Institute of Microbiology, Friedrich Schiller University Jena, Jena, Germany; Estacion Experimental del Zaidin, SPAIN

## Abstract

Plants interact with a wide variety of fungi in a mutualistic, parasitic or neutral way. The associations formed depend on the exchange of nutrients and signalling molecules between the partners. This includes a diverse set of protein classes involved in defence, nutrient uptake or establishing a symbiotic relationship. Here, we have analysed the secretomes of the mutualistic, root-endophytic fungus *Piriformospora indica* and *Arabidopsis thaliana* when cultivated alone or in a co-culture. More than one hundred proteins were identified as differentially secreted, including proteins associated with growth, development, abiotic and biotic stress response and mucilage. While some of the proteins have been associated before to be involved in plant-microbial interaction, other proteins are newly described in this context. One plant protein found in the co-culture is PLAT1 (Polycystin, Lipoxygenase, Alpha-toxin and Triacylglycerol lipase). PLAT1 has not been associated with plant-fungal-interaction and is known to play a role in abiotic stress responses. In colonised roots PLAT1 shows an altered gene expression in a stage specific manner and *plat1* knock-out plants are colonised stronger. It co-localises with Brassicaceae-specific endoplasmic reticulum bodies (ER-bodies) which are involved in the formation of the defence compound scopolin. We observed degraded ER-bodies in infected Arabidopsis roots and a change in the scopolin level in response to the presence of the fungus.

## Introduction

The roots of more than 80% of all land plants form mycorrhizal interaction [1]. The interactions can shift from mutualism to parasitism depending on environmental conditions and the communication between the organisms [2]. The communication between the symbionts is driven by molecules released into the rhizosphere by both partners including diverse protein classes [3–6]. Based on genome analyses, it has been estimated that the proportion of released fungal proteins represent about 3% to 10% of the corresponding total proteome [6]. Furthermore, the size of the predicted secretomes correlates with the lifestyle of a fungus. For example, pathogenic fungi appear to have more secretory proteins compared to saprophytic and mutualistic species [6, 7]. Proteomic-based technologies and bioinformatic tools identified multiple fungal proteins which determine the mode of interaction and reprogram the root cells [8–10]. Depending on the organism and strain colonising the plant, the number and amount of the secreted proteins from both partners can vary, as shown during the symbiotic interaction of different fungi with *Pisum sativum*, *Medicago truncatula* and *Nicotiana benthamiana *[11–14]. Mutualistic fungi secrete also various effector proteins that repress plant defence responses. After entering the plant cell, some effector proteins are transported to the plant nucleus, where they can interact with transcription factors and repress plant defence [15]. This is well studied for the small secreted protein 7 (SP7) from *Rhizophagus irregularis* and MISSP7 (mycorrhiza-induced small secreted protein-7) from *Laccaria bicolor* [16–18].

Similar to the fungal secretome, between 6% to 17% of plant proteins are estimated to be secreted [19, 20]. These proteins have multiple functions including growth regulation, communication, nutrient uptake and defence responses. However, most of them are uncharacterised [21, 22]. Among the better studied are germin-like proteins which can produce reactive oxygen species in response to stress and during plant growth. They have multiple roles outside the cell, particularly in defence and development [23, 24]. Furthermore, small peptides, such as members of the CLE (clavata3/embryo-surrounding region) family, have been implicated in development, inter-plant communication, defence and in the formation of new symbiosis related organs such as nodules [21, 25, 26].

Exudate proteins are often identified by bioinformatic tools based on the prediction of their N-terminal secretion sequence which directs them into the endoplasmic reticulum (ER) from where they are secreted via vesicle fusion with the plasma membrane [16, 27, 28]. However, only ~ 50% of the known secreted plant proteins possess such a signal [11, 22] whereas others utilise unconventional mechanisms of secretion, which resemble those of animal exosomes [29] or include exocyst-like structures [30]. For example, between 50% to 80% of proteins found in the secretome of Arabidopsis after treatment with salicylic acid or pathogens do not possess a N-terminal secretional sequence [11, 31, 32]. Although some of these proteins can also be contaminations, e.g. due to cell damage, untargeted secretome studies are valuable addition to bioinformatic-based studies. While *A*. *thaliana* does not form mycorrhizal associations, the plant can be colonised by endophytes, such as *Piriformospora indica*. This fungus of the order Sebacinales was isolated from roots of plants growing in the Indian Thar desert [33]. The fungus has a saprophytic lifestyle and colonises the roots of many plant species. Colonised plants produce more biomass, in particular under unfavourable conditions, and are more resistant to abiotic and biotic stresses [34–36]. Based on expression profiles during the colonisation of Arabidopsis [37] and barley roots [5], the interaction can roughly be split into three stages, which corresponds to the saprophytic and mutualistic traits of *P*. *indica* [38]. The initial contact is characterised by the down-regulation of plant defence mechanisms, followed by a phase in which the fungus reduces the expression of genes associated with saprophism [38]. During the last phase, a long-lasting beneficial interaction is established [5, 12, 37].

To balance beneficial and non-beneficial traits in a symbiosis, the propagation of microbes in the roots must be controlled by host defence compounds. One of them is the coumarin scopoletin, which accumulates in the ER-body [39]. The ER-bodies derive as subdomains from the ER network and have specialised functions in glucosinolate-based defence [39–41]. ER-bodies harbour high levels of β-glucosidases including PYK10, the most abundant enzyme of the organelle [42]. When ER-bodies are disrupted during pathogen attack or damage, PYK10 and other enzymes are released into the cytoplasm. PYK10 is involved in establishing the interaction between *P*. *indica* and *A*. *thaliana* roots [43]. The roots of mutant lines with reduced PYK10 level are overcolonised by *P*. *indica*, although *PYK10* expression is unaffected by *P*. *indica* in wild type (WT) seedlings [43, 44]. PYK10 hydrolyses multiple indole glucosinolates and scopoletin to scopolin [44, 45]. Scopoletin, and its glycone scopolin, accumulates in roots and helps plants to fine tune their defence responses by scavenging of reactive oxygen species [46, 47]. Scopoletin acts as antifungal component against *Alternaria alternata* in tobacco and is strongly suppressed in the beneficial symbiosis of tomato with *R*. *irregularis* [48, 49]. A key enzyme for scopoletin biosynthesis is the feruloyl-CoA 6'-hydroxylase 1 (F6'H1), and the accumulation of this secondary metabolite is severely reduced in the corresponding knock-out line [46]. Interestingly, biosynthesis of scopoletin can also be stimulated by ectopic expression of the Arabidopsis PLAT1 and -2 (Polycystin, Lipoxygenase, Alpha-toxin and Triacylglycerol lipases) proteins in tobacco [50]. It has been hypothesized that the PLAT domain provides a docking platform for proteins to regulate their catalytic activities [51–53]. AtPLAT1 and -2 proteins are functionally and physically associated with ER-bodies [51]. This suggests an interaction with PYK10, although the exact function of the PLAT domain is still enigmatic.

In this study, we identified proteins which are found in the growth medium of *A*. *thaliana*, *P*. *indica* or *P*. *indica* co-cultivated with *A*. *thaliana*. Proteins which are only found in the co-culture or disappear from it, are considered symbiosis-specific and are mainly involved in growth and defence-related processes. Many enzymes belonging to the latter category are implicated in the production or release of secondary metabolites. The role of one of these proteins, PLAT1, was investigated in more detail and its postulated function in the formation of the defence compound scopolin was related to the degradation of ER-bodies and root colonisation.

## Results

### Secretome analysis of *P*. *indica* and *A*. *thaliana* reveal distinct changes in the co-culture

To identify symbiosis-specific proteins that are secreted during the early phase of the interaction between *P*. *indica* and *A*. *thaliana*, we co-cultivated both organisms in liquid plant nutrition media (PNM) for three days and compared them with cultures of the plant and fungus grown separately (S1 Fig). The spent media were filtered and proteins were isolated by solid phase extraction followed by LC-MS/MS (liquid chromatography–tandem mass spectrometry) analysis. The resulting MS/MS spectra were searched against the UniProt database (http://www.uniprot.org/) [54] where a total of 590 different *A*. *thaliana* and 164 *P*. *indica* proteins were identified in three independent biological replicates (S1 Table). In the single cultures of Arabidopsis 199, 316 and 117 proteins were observed within three replicates, while the number of identified proteins in the single fungal cultures were 39, 76 and 76. In the co-culture 140, 311 and 85 plant proteins and 120, 108 and 84 fungal proteins were observed per replicate. The Venn diagram in S2A Fig shows the overlap of Arabidopsis proteins in the co-culture among all replicates. 57 plant proteins were common to all three replicates, which represent 16% of all identified proteins. In total, 33% of proteins were found in at least 2 replicates (S2A Fig). When comparing the replicates of the plant and fungal single cultures, similar percentages were found.

**Fig 1:**
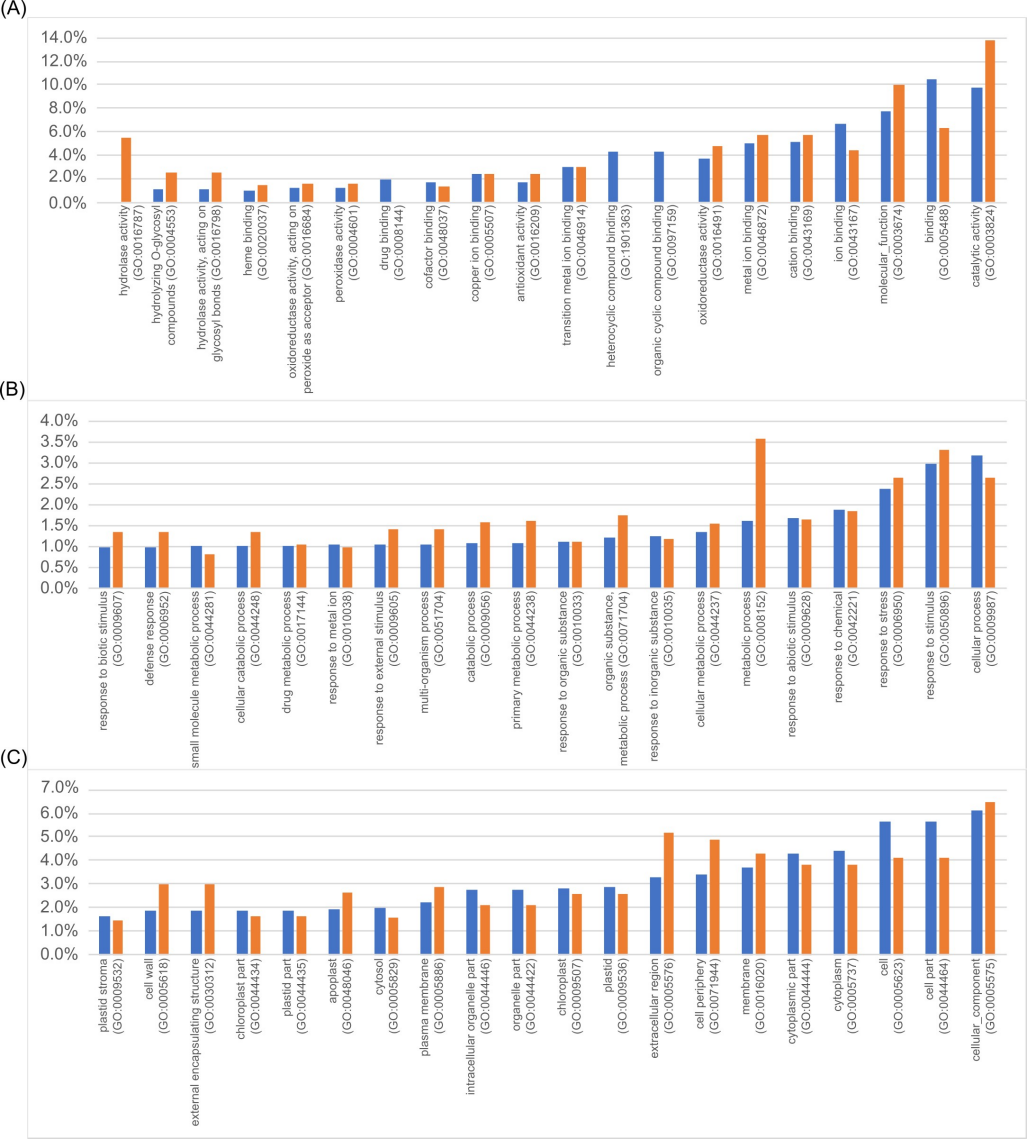
GO enrichment analysis of the secretomes. All identified plant proteins were analysed using the GO term analysis tool using AmiGO (http://www.geneontology.org, version 1.8) and sorted according to the categories of biological processes (A), molecular functions (B) and cellular components (C). The 20 most abundant GO terms are presented here (in percentage). Blue bars indicate the frequency of this GO term in single cultures, orange bars indicate the frequency of the respective GO term in co-cultures.

For further investigations, we took all identified plant proteins and classified them (S2 Table) according to their GO (Gene ontology) terms using AmiGO (http://www.geneontology.org, version 1.8) [55–57]. Due to the absence of a functional annotation for a high number of fungal proteins, no GO terms have been captured for *P*. *indica*. The GO terms annotated to plant proteins were sorted within the categories “molecular functions”, “biological processes” and “cellular components” (Fig 1A–1C). For each category, the 20 most abundant terms are shown in Fig 1A–1C. The functional categorisation revealed that secreted plant proteins in both cultures belong mainly to identical GO terms, however, the number of protein entries varies for each term. As shown in Fig 1A, the most frequent term (10%) related to proteins found in the single culture of Arabidopsis is “binding of different compounds” (GO:0005488), whereas the most abundant term related to proteins from the co-culture (12%) is “catalytic activity” (GO:0003824). As another example, about 5% of the proteins in the co-culture play a role in hydrolysis (GO:00016787), while proteins associated with this GO term were not found in the single culture. Among the terms of the category “biological processes” (Fig 1B), slight differences between the single and co-culture were observed. For instance, proteins with the GO term “metabolic process” (GO:0008152) were found twice as often in the co-culture. Furthermore, the terms “defence”, “external stimuli”, “response to biotic stimulus” and “catabolic process” were found more often for co-cultures. The category “cellular component” (Fig 1C) included notably more proteins associated with the GO terms “extracellular region” and “cell periphery” in the co-culture. Contrary, GO terms associated with “cellular processes” or “organelles” were more frequently found in the single culture.

#### *A*. *thaliana* responds to the presence of *P*. *indica* by an altered secretion of proteins associated with defence and growth

**Fig 2:**
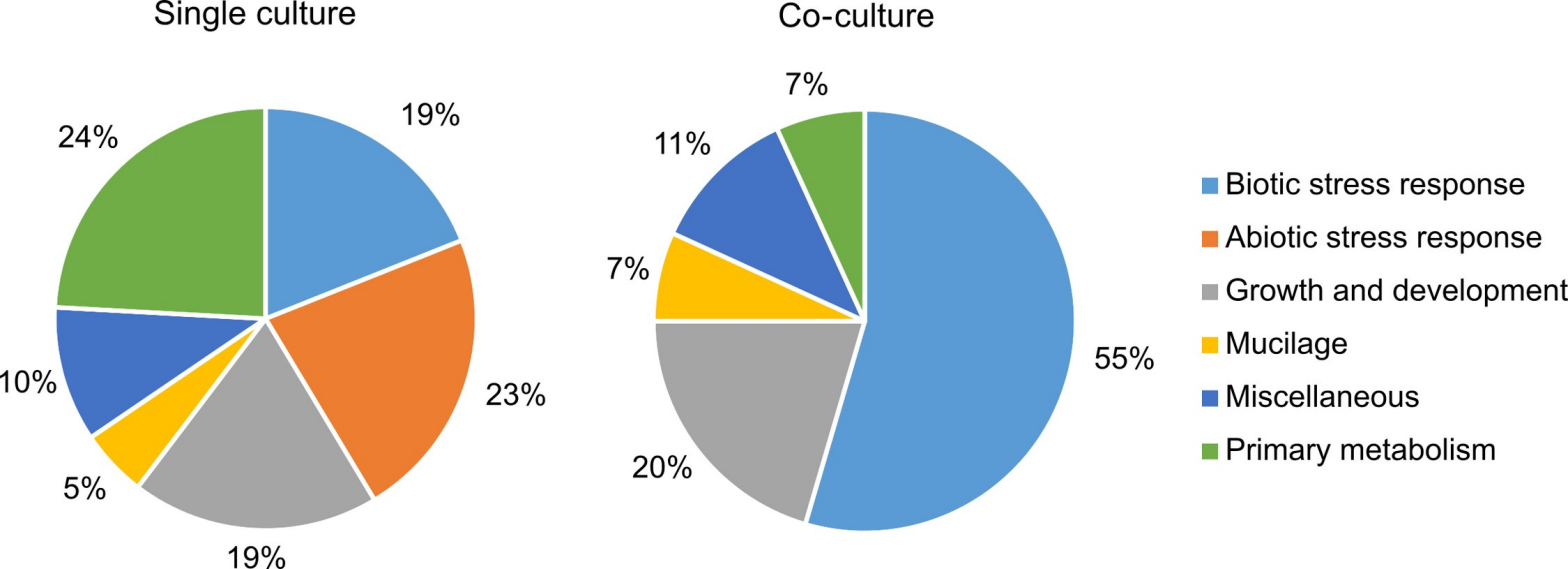
Differentially secreted proteins. Arabidopsis proteins that were found in at least two replicates and with different frequencies in the single and co-culture (S2 Table) were sorted according to their primary function. Numbers are given in %.

To better understand changes in the composition of secreted proteins, we looked for specific differences between the co-culture and single culture (S2 Table). The analysis considered only proteins found in two or more root exudates and these proteins were classified based on their function. We observed 102 proteins to be differentially secreted (Fig 2). The most enriched proteins in the single culture are associated with the functions “primary metabolism”, “abiotic stress response”, “growth and development” and “biotic stress response” (~20% each). In the co-cultures, 55% of the proteins play a role in defence against biotic stresses, 20% have been described in the context of growth and development, 11% belong to the primary metabolism and 7% proteins are related to abiotic stress responses. Furthermore, 11% proteins are classified as miscellaneous. Interestingly, 7% of the differentially secreted proteins are associated with mucilage.

Among proteins, which were involved in development and growth and which were predominantly detected in the co-culture, are the ribonuclease T2 (At1G14220), the lipid recognition protein At5G23840 and the pectin methylesterase inhibitor At1G23205. Examples related to biotic stress responses include the pathogenesis-related protein 5 (At1G75040), which was strongly induced by the fungus. In Arabidopsis, it has been implicated in systemic acquired resistance [58]. Furthermore, the two functionally distinct germin like proteins (GLPs), GLP2 (At1G02335) and GLP4 (At1G18970) appear to be secreted into the co-culture upon interaction with the fungus. Additionally, PLAT1 (At4G39730) was found in the medium of the co-culture.

The single cultures contain a different set of proteins. The list includes several proteins associated with abiotic stress responses, particularly some proteins involved in cadmium stress such as At1G11860 (aminomethyl transferase), At1G48030 (dihydrolipoyl dehydrogenase 1) and At3G23990 (chaperonin CPN60) [59]. The single culture also contains ABA (abscisic acid)-inducible proteins such as At1G76180 (dehydrin ERD14), At1G20440 (dehydrin COR47), At5G15970 (stress-induced protein KIN2), the glycine-rich protein At1G04800 and the peroxidase 69 (At5G64100) [60–69]. Surprisingly, the well-known defence compound remorin 3 (At2G45820), which was shown to be associated with plasma membranes and to bind proteins derived from pathogens [70–72], was only detected in the single cultures. Interestingly, the analyses also detected six mucilage-related Arabidopsis proteins as potential targets for *P*. *indica* (S2 Table). In the co-culture, the mucilage-related proteins aspartic protease 1 (At3G18490), responsive to dehydration 21A (At1G47128) and its homologue 21B (At5G43060) were identified [73]. In contrast, the xyloglucan endotransglucosylase 6 (At5G65730) was only found in the single culture. A homologue of endotransglucosylase 6 was found in the mucilage of maize roots [74]. Also, the oxalate-CoA ligase At3G48990, which has a role in seed mucilage, and the subtilisin-like protease 1.8 (SBT1.8, At2G05920) were only found in the single culture [73, 75]. The latter is highly homologues to SBT1.7 (At5G67360), a key protein for mucilage formation, which is present in both the single and the co-culture (S1 Table). Other mucilage-associated proteins such as fasciclin-like arabinogalactan protein 10 (At3G60900), auxin-induced in root cultures protein 12 (At3G07390) and non-specific lipid-transfer protein 6 (At3G08770) were found in both fractions (S1 Table) [73].

All differentially enriched proteins were also examined individually to assess whether they possess an N-terminal secretion sequence using available bioinformatics tools TargetP 1.1 (http://www.cbs.dtu.dk/services/TargetP/), SignalP 4.1 (http://www.cbs.dtu.dk/services/SignalP/) and Predotar 1.3 (https://urgi.versailles.inra.fr/Tools/Predotar) [76, 77]. The results showed that 36 out of 44 proteins in the co-culture and 18 out of 58 proteins in the single culture possess a predicted N-terminal secretion sequence (S2 Table).

#### Symbiosis reprograms the *P*. *indica* secretome

A total of 164 *P*. *indica* proteins were found in all experiments (S1 Table): 36 of them were differentially enriched between the treatments (S2 Table). Among these proteins, two were found predominantly in the single fungal culture (S2 Table): PIIN_00867, an uncharacterised protein, -and PIIN_06517 related to glyoxal oxidase. 34 proteins were present only in the co-culture. Generally, many of the fungal proteins identified in this study are uncharacterised or possess homology to degrading enzymes. For instance, PIIN_01733, which was found in the co-cultures, is a putative beta-mannanase with homology to hydrolases from two saprophytic fungi [78]. Additionally, we analysed fungal proteins for subcellular localization using available bioinformatics tools: 21 contained an N-terminal secretory signal peptide (S2 Table). One of them is a indole-3-acetaldehyde dehydrogenase (PIIN_04899) which has been suggested to be involved in the formation of auxin [79].

#### *P*. *indica*-root colonisation influences *PLAT1* and leads to changes in scopolin production

**Fig 3:**
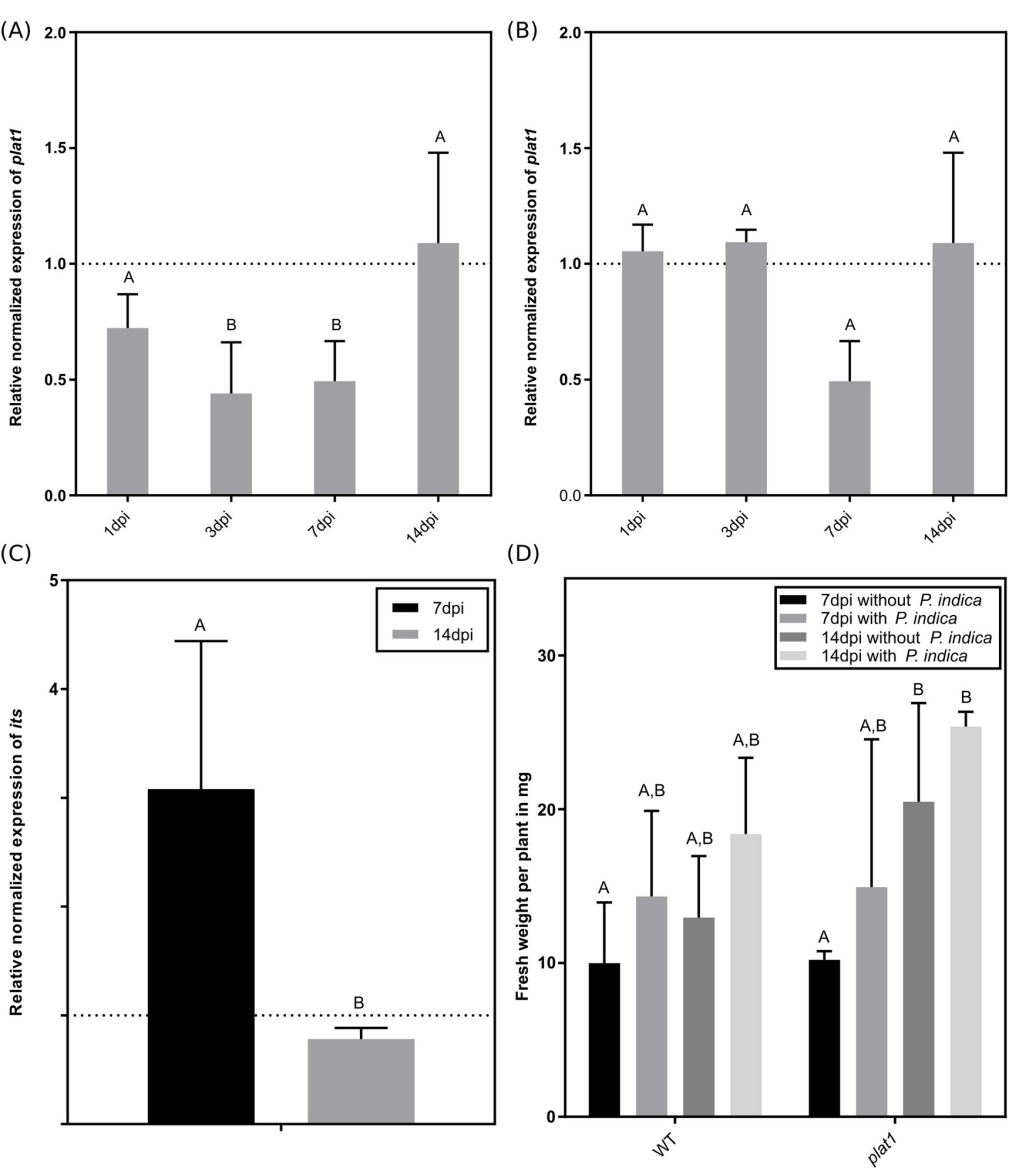
Characterisation of *plat1* mutant and *plat1* gene expression. Gene expression of *PLAT1* in WT roots (A) and leaves (B) was determined between one day and 14 days of colonisation. The dashed line represents levels of mRNA in uncolonised WT roots. (C) Colonisation of *P*. *indica* in WT and *plat1* roots determined by RT-qPCR. The dashed line represents the level of fungal internal transcribed spacer (*its*) mRNA in colonised WT roots after one and two weeks, respectively. At 7dpi (days post inoculation) the *plat1* mutant was significant stronger colonised compared to WT plants. (D) Effect of *P*. *indica* on fresh weight in WT and different mutant *A*. *thaliana* plants after one and two weeks of colonisation. All experiments were repeated three to six times with at least ten plants each. Error bars represent standard deviation. Different letters represent significant differences between treatments at p<0.05 (Two-Way ANOVA, followed by a Bonferroni correction).

As a case example to study the secretome, we further analysed the role of the Polycystin, Lipoxygenase, Alpha-toxin and Triacylglycerol lipases 1 (PLAT1). This protein was found in the medium of the co-culture and has not been reported in context with *P*. *indica* before. We checked the expression level of *PLAT1* in colonised WT roots and observed a significant lower level of *plat1* mRNA at three and seven days of co-cultivation. After two weeks the level of *plat1* mRNA was comparable to uncolonised plants (Fig 3A). In shoots no regulation was observed (Fig 3B). A weak but not significant downregulation was observed for the homologous *plat2* mRNA, whereas for the putative pseudogene *plat3* no regulation was observed. Interestingly, a ten-time higher expression in leafs compared to roots was observed for *plat3*. Nevertheless, for all tested time points and plant parts the expression level of *plat3* was extremely low (S2B and S2C Fig). Since *P*. *indica* had the strongest effect on *PLAT1* expression, we took a closer look at the colonisation of *plat1* mutant seedlings by the fungus. After one week, we found a three times higher amount of fungal RNA in the *plat1* mutant compared to the WT seedlings, whereas after two weeks no differences in colonisation between *plat1* mutants and WT plants was observed (Fig 3C). This stronger colonisation did not have an impact on the total fresh weight of the *plat1* mutants (Fig 3D).

**Fig 4:**
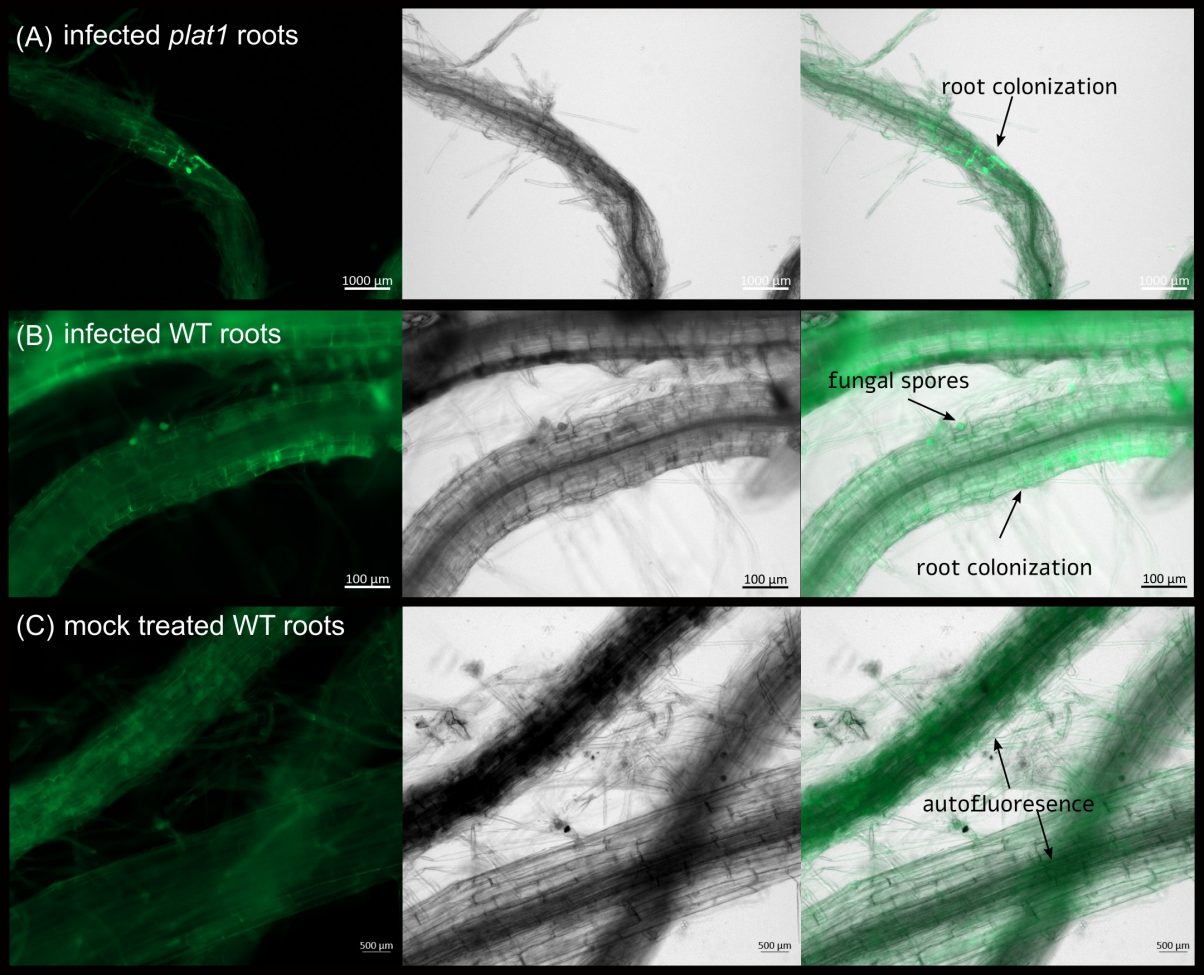
Root colonisation of Arabidopsis WT and *plat1* by *P*. *indica* at 7 dpi. Colonisation of Arabidopsis *plat1* (A) and WT roots (B) was imaged using fluorescence microscopy at 7 dpi. Uncolonised roots are shown at (C). The first pictures show the autofluorescence of the root and GFP (green fluorescent protein) fluorescence of *P*. *indica*. The second pictures show the bright field image. The last pictures show the overlay of all images for each row.

These results could be confirmed by microscopy (Fig 4): after seven days we observed a stronger colonisation in the *plat1* mutant (Fig 4A) compared to the WT (Fig 4B), where almost no fungus could be detected.

Since AtPLAT1, which was identified in the co-culture, promotes scopoletin biosynthesis [50], and an upregulation of scopoletin in colonised *A*. *thaliana* roots has been described before [12], we analysed the connection between PLAT1, scopoletin and *P*. *indica* colonisation.

**Fig 5:**
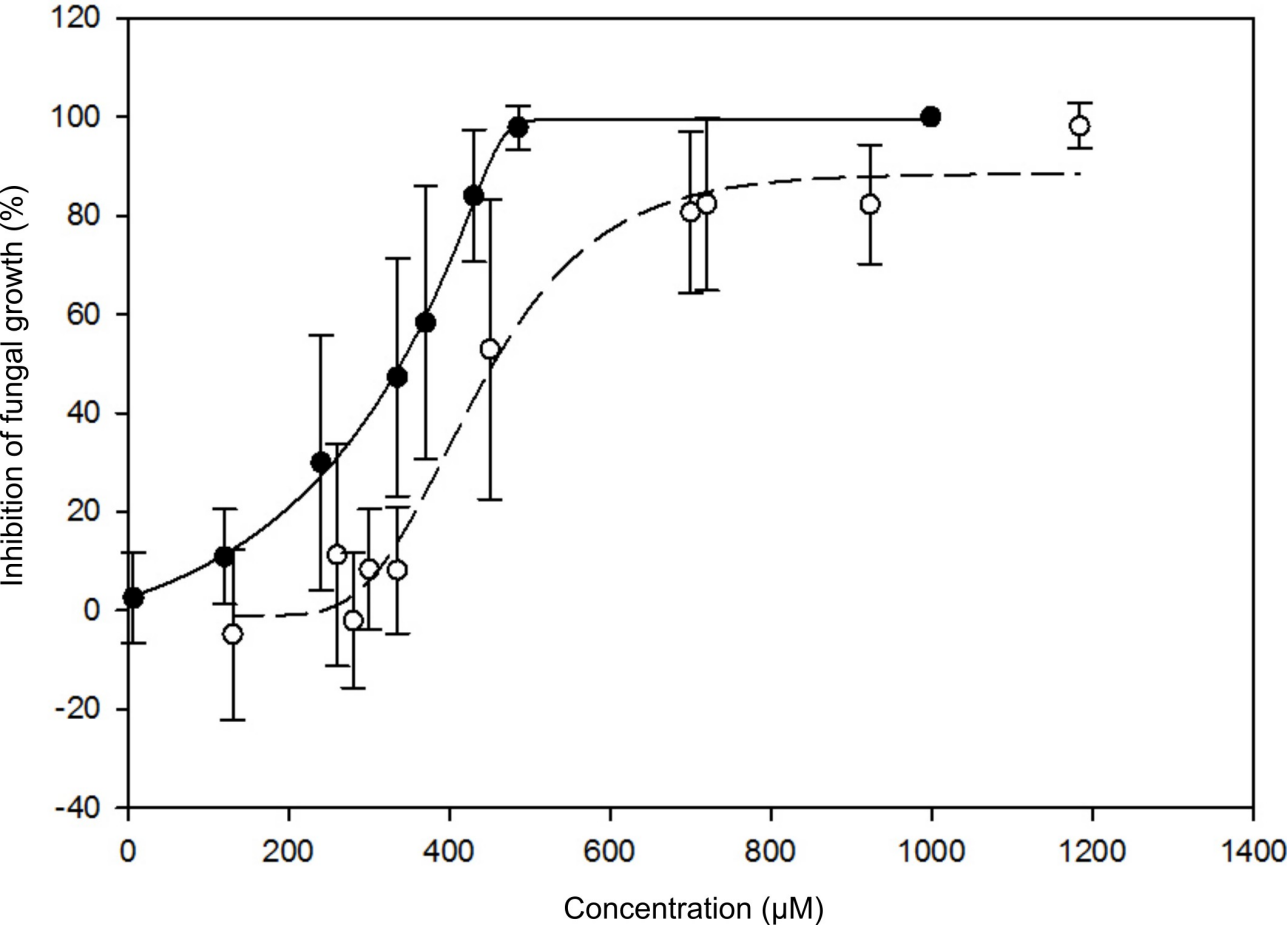
Growth inhibition of *P*. *indica* by scopoletin / scopolin. Sigmoidal regression line of the growth effect of scopoletin (full dots) and scopolin (hollow dots) on growth of *P*. *indica*. Error bars represent standard deviation. The experiment was repeated in three biological replicates with two technical replicates each.

In a first step we examined the influence of scopolin and scopoletin on *P*. *indica in vitro* and found that both chemicals inhibit the growth of *P*. *indica* with a half maximal inhibitory concentrations (IC_50_) of 0.34 mM and 0.45 mM, respectively (Fig 5).

**Fig 6:**
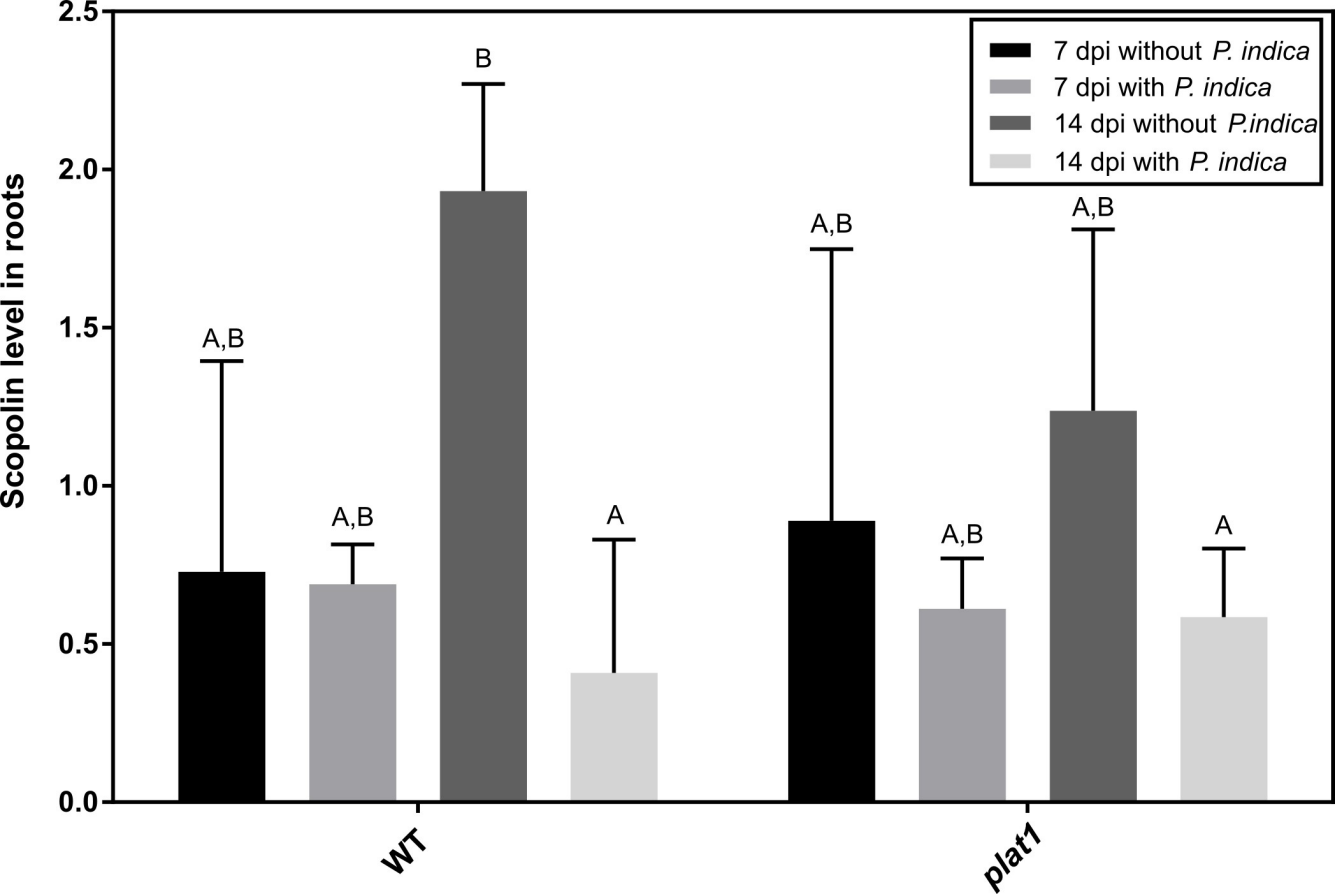
Characterisation of *plat1* mutant and *plat1* gene expression. First, we determined the scopolin levels in root of WT and *plat1*, and the peak areas of HPLC chromatograms were normalised by an internal standard. All experiments were repeated four to five times with at least ten plants each. Error bars represent standard deviation. Different letters represent significant differences between treatments at p<0.05 (Two-Way ANOVA, followed by a Bonferroni correction).

Scopolin is much more abundant than scopoletin and can easily be measured in high quantities, in Arabidopsis roots [80]. Therefore, we studied the influence of *P*. *indica* on scopolin production in Arabidopsis roots *in vivo*. After two weeks, the mock treated WT roots showed a four-time upregulation of scopolin compared to the other time points, whereas in colonised roots the scopolin level was not altered. Instead, the same level of scopolin was observed for all time points and treatments in the *plat1* mutant (Fig 6).

**Fig 7:**
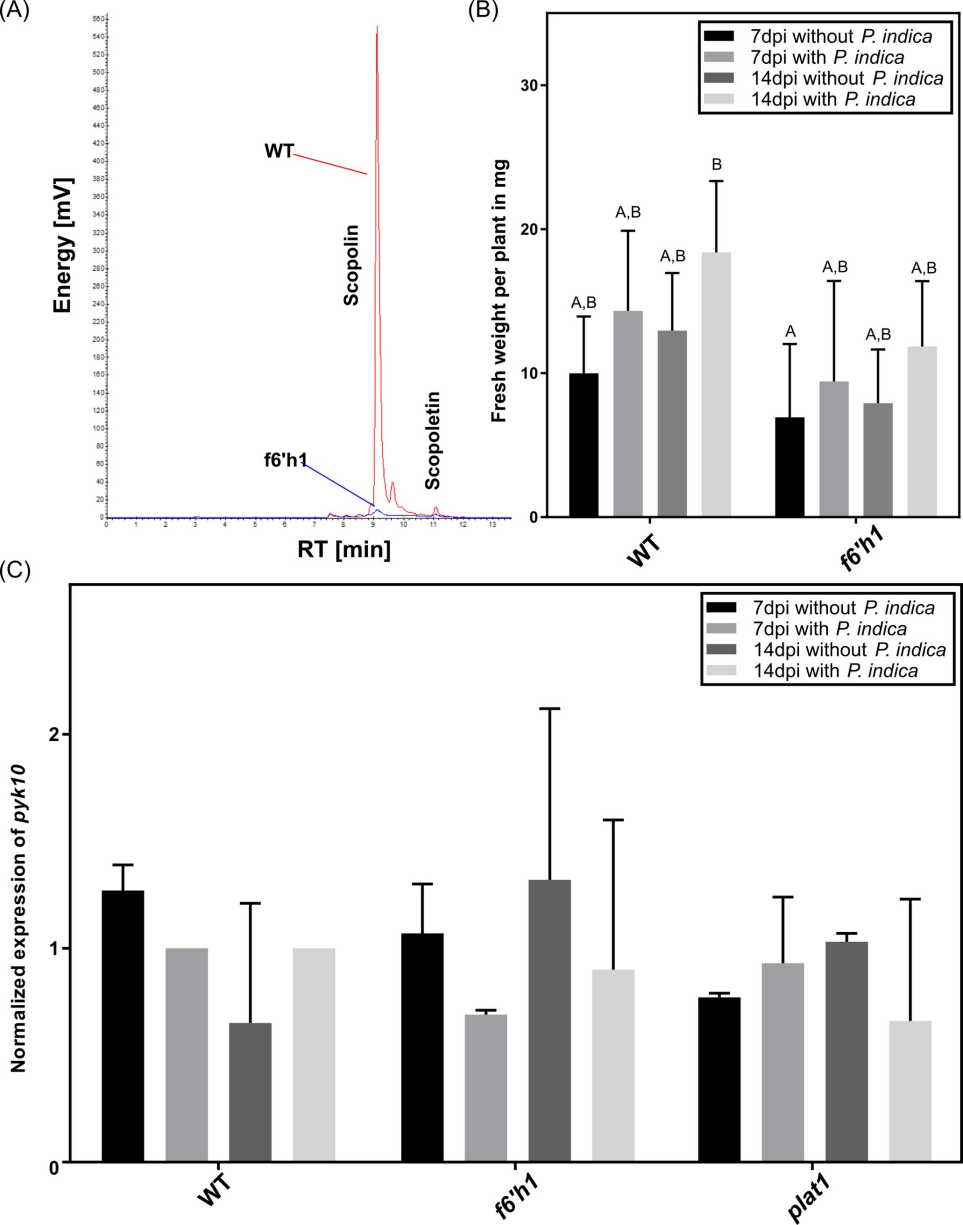
Characterisation of the *f6’h1* mutant. (A) Fluorescence (336/438 nm) HPLC chromatograms of root extracts from WT (red) and *f6'h1* (B) Growth promotion of WT and *f6’h1* by *P*. *indica* after 7 and 14 days of co-cultivation. At 14 dpi a slightly higher growth-stimulating effect was visible for *f6’h1* compared to the WT. (C) Expression of *PYK10* in the roots of WT and *f6'h1* mutants 14 days after co-cultivation with or without *P*. *indica*. All experiments were repeated three to four times with at least ten plants each. Error bars represent standard deviation. Different letters represent significant differences between treatments at p<0.05 (Two-Way ANOVA, followed by a Bonferroni correction).

To examine whether scopolin alone is responsible for the altered colonisation in the *plat1* mutant, we investigated the performance of the fungus in the *f6’h1* Arabidopsis mutant line where scopolin and scopoletin production is severely reduced (Fig 7A). When comparing the fresh weight between WT and *f6’h1* plants, we observed, similar to *plat1* (Fig 3D), that the mutant lines are not influenced by the presence of the fungi. Furthermore, the mutant lines were smaller than WT plants (Fig 7B). Since PYK10 can be involved in scopolin formation, we checked its expression: *pyk10* is not influenced by the fungus neither in the WT plants nor in the *plat1* or the *f6’h1* mutant background (Fig 7C). Other genes, which are also associated with scopolin formation, are not influenced by the fungus in *f6’h1* or WT plants as well (S3 Fig).

**Fig 8:**
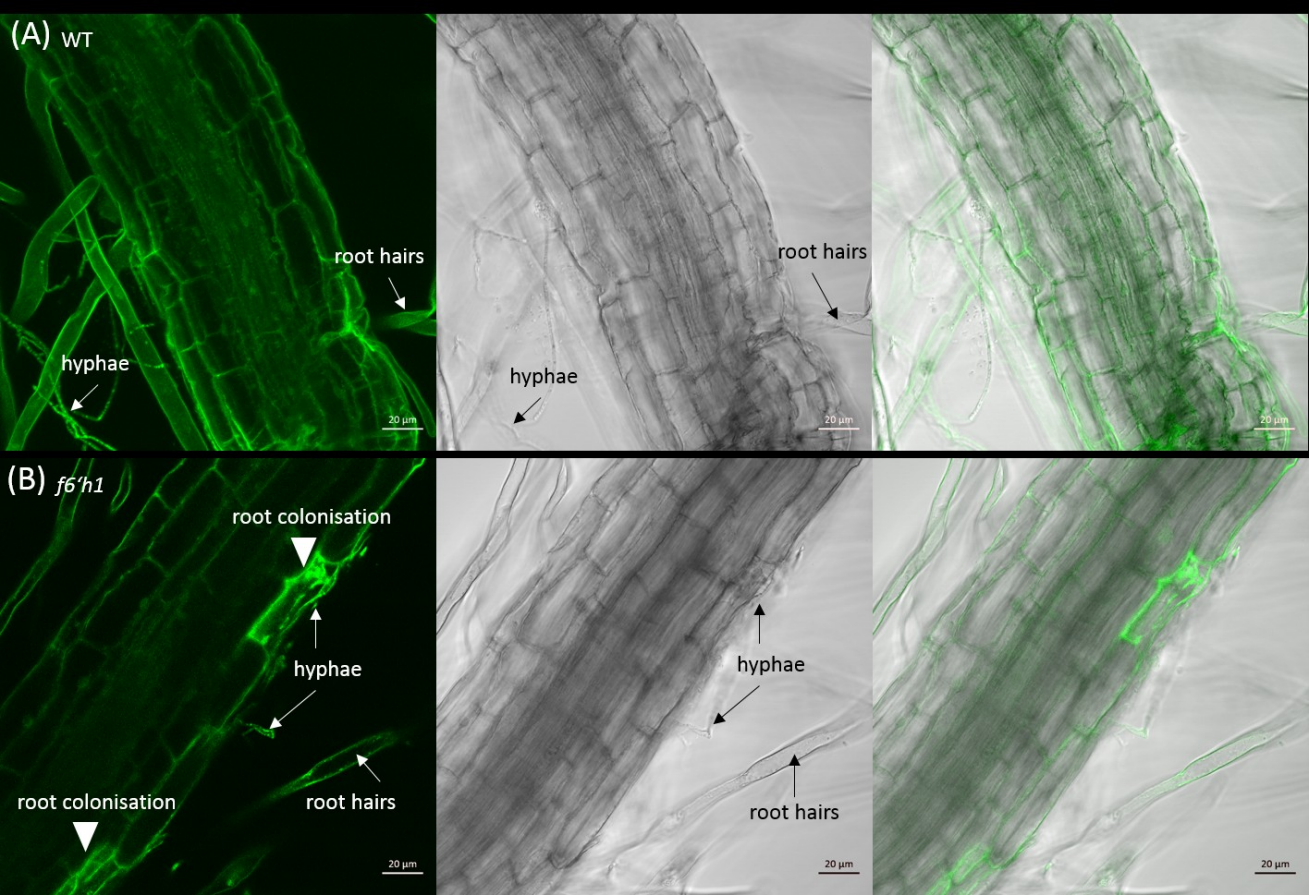
Root colonisation of Arabidopsis WT and *f6’h1* by *P*. *indica* at 14 dpi. Arabidopsis WT (A) and *f6’h1* (B) were imaged using confocal microscopy. The first pictures show the autofluorescence of the root and GFP fluorescence of *P*. *indica*. The second pictures show the bright field image. The last pictures show the overlay of all images for each row.

We further analysed the colonisation of Arabidopsis roots by microscopy. Seven days after co-cultivation most of the hyphae associated with WT roots were detected on the root surface between the epidermal and subepidermal root cells and barely detectable within root cells, whereas in *f6’h1* mutant roots, hyphae could be detected around and within root cells. This effect was even stronger 14 and 19 days after co-cultivation (Fig 8).

#### *P*. *indica*-root colonisation causes degradation of ER-bodies

**Fig 9:**
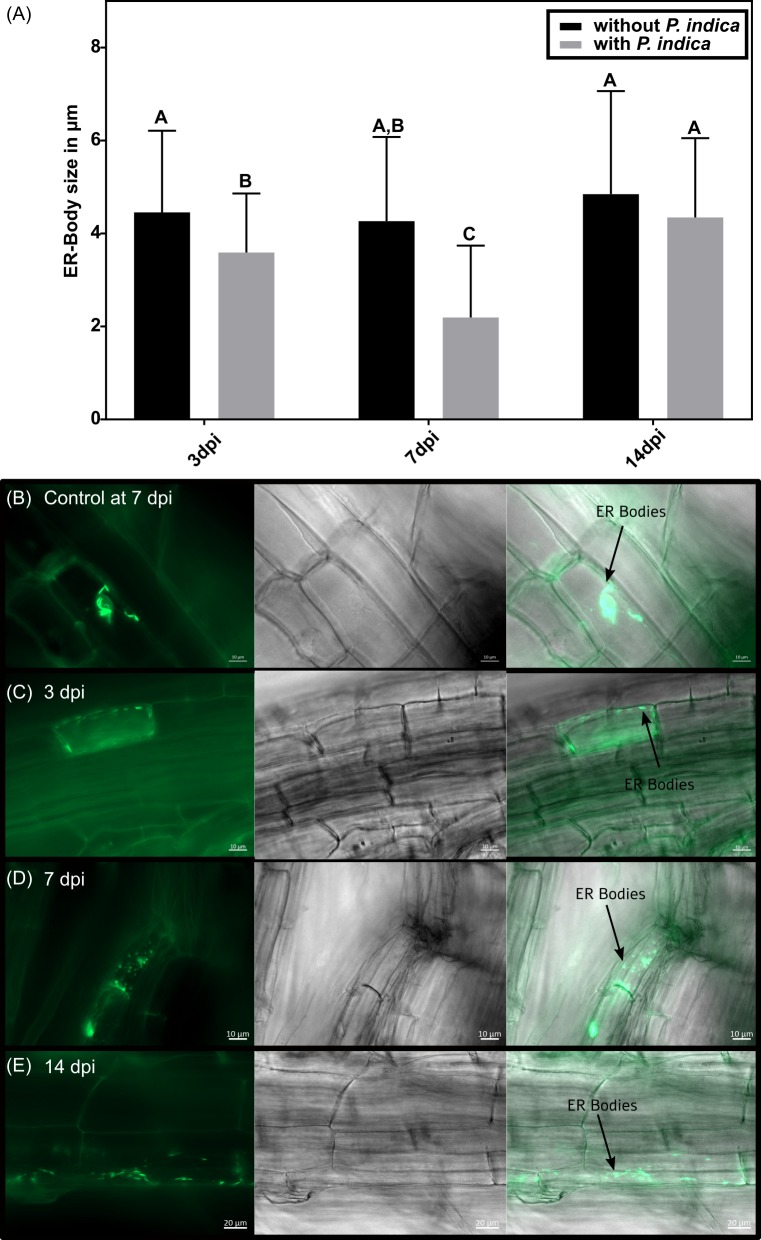
Effect of *P*. *indica* on ER-body size. (A) Comparison of the ER-body length in Arabidopsis root cells without and with *P*. *indica* at 3 dpi, 7 dpi and 14 dpi. Error bars represent standard deviation. Different letters represent significant differences between treatments at p<0.05 (Two-Way ANOVA, followed by a Bonferroni correction). Per time point and treatment four roots were used and the size of 20–100 ER-bodies per root was measured. (B) Fluorescence microscopy of ER-bodies in *A*. *thaliana* root without *P*. *indica* at 7 dpi and with *P*. *indica* at 3 dpi (C), 7 dpi (D) and 14 dpi (E).

The Arabidopsis lipases PLAT1 and -2 are functionally associated and co-localise with ER-bodies *in vivo* [51]. Thus, we analysed the fate of ER-bodies during the establishment of symbiosis (Fig 9). The size of ER-bodies in uncolonised roots is not altered during the development of the plant but decreases during the interaction with the fungus (Fig 9A). After three days, the ER-bodies in the WT have the normal rod-shaped structure (Fig 9B) but are slightly smaller in the colonised roots (Fig 9C). After seven days, the ER-bodies in the fungal treated roots have a significant reduced size and, moreover, a diffuse signal was observed (Fig 9D). This effect diminishes after two weeks and the size and structure of the ER-bodies was again comparable to the mock treated samples (Fig 9E).

We further determined the size of ER-bodies in the liquid culture at the time point of the secretome study. No difference in structure was observed between the mock treated and colonised roots (S4 Fig). But when comparing the liquid culture with the solid culture, a slight size-reduction was observed for the ER-bodies in untreated roots at 3 dpi (S5 Fig).

## Discussion

### *P*. *indica* alters the secretome of colonised plants

The availability of the complete genome sequence and a system of hydroponic cultivation makes *P*. *indica* an interesting model endophyte for studying the molecular basis of beneficial symbiotic interactions. In this study, *P*. *indica* and *A*. *thaliana* were cultivated alone and together in hydroponic media and the secreted proteins were compared after three days of co-cultivation. This approach allowed us to identify symbiosis-relevant proteins secreted during the early stage of interaction. At the time point of this study, the fungus and the plant have not established a mutualistic symbiosis and the fate of the interaction is still undetermined [12, 38, 81]. Our results demonstrate that 102 plant proteins were differentially enriched the presence of the fungus (S2 Table and Fig 2). The most severe alterations in the level of plant proteins affect biotic and abiotic stress responses, the primary metabolism and mucilage synthesis.

In the single culture, proteins belonging to the primary metabolism (24%), abiotic stress (23%), growth and development (19%) and biotic stress (19%) were present with a similar frequency. On the contrary, in the co-culture proteins involved in biotic stress responses (55%) as well as growth and development (20%) were more abundant. The high proportion of proteins associated with these GO terms is not surprising, as *P*. *indica* influences many aspects of plant growth and defence mechanisms [5, 34, 37, 81, 82].

Abiotic stress response was one of the most dominant categories in the single culture but not the co-culture. Interestingly, we identified several proteins associated with cadmium stress in the single culture. It is unclear whether these proteins play a direct role in cadmium stress responses or whether they have additional function, but intriguingly, for tobacco and sunflower it was reported that *P*. *indica* alleviate cadmium specific stress [83].

While abiotic stress, such as osmotic stress, can be expected in a liquid cultivation system, we were surprised to find almost no proteins which play a role in this category in the co-culture. As exception, the abiotic stress-related cysteine proteinase RD21A, the Aspartic Protease in Guard Cell 1 and PLAT1 were identified in the co-culture [73]. PLAT1 is induced by ABA and plays a role in abiotic stress response, but our and previous findings indicate an additional role of PLAT1 in plant-microbial interaction [50]. ABA plays a vital role in the interaction of plants with *P*. *indica*. It accumulates stage-specific in colonised roots, which is required for successful colonisation [81, 84]. Thus, an altered level of ABA induced by the fungus may be involved in or responsible for the observed differences in PLAT1 accumulation.

Besides PLAT1, we identified 34 differentially regulated proteins with a role in biotic stress. Some of the proteins have already been described in the context of *P*. *indica* colonisation before. For example, the polygalacturonase inhibitor 1, which restricts programmed cell death of the host [85], was found in the co-culture. A homologue of this protein was recently identified as a target of *P*. *indica* in Chinese cabbage [86]. Another protein from the co-culture, the pectin methylesterase inhibitor At1G23205 is induced by volatiles from a plant growth-promoting *Bacillus subtilis* strain [87].

In a recent proteome study, *Brassica napus* was co-cultivated with *P*. *indica*. Similar to our study, proteins associated with biotic stress as well as growth were identified. Another investigation showed that a GLP was downregulated in rape by the fungus after 28 days of co-cultivation [88]. In our short-time assay, homologues of this protein, GLP2 and GLP4, were found in the medium of the co-culture. While GLP4 has an important part in controlling *Blumeria graminis* infection in wheat and barley [89, 90], GLP2 plays a role in root development, possibly by controlling the distribution of carbohydrates between different root parts [91]. These findings are not surprising since the roots are in contact with different fungal cell wall components and the fungus might manipulate the carbohydrate metabolism in roots to stimulate plant growth [92]. Furthermore, the beginning of interaction is characterised by a stressful biotrophic-like phase [81]. At the early time point studied here, the fungus establishes the colonisation, which is accompanied by the balanced regulation of defence compounds. Interestingly, we found that the salicylic acid level is reduced while jasmonate, indolic glucosinolates and flavonoids are increased [12, 37]. Apparently, after 3 days of co-culture, jasmonate-dependent defence strategies dominate in the co-culture which further support the concept that a beneficial interaction phase has not yet been reached.

Another observation was that proteins of the primary metabolism were found predominantly in the single culture and not the co-culture (24% and 7%, respectively). Since secreted proteins were analysed, this observation is difficult to explain. Based on the results discussed above, one would expect that the fungus represses the primary metabolism to shift metabolites towards defence during the early time point investigated in this study. Under this assumption the amount of both cellular and exudated proteins involved in the plant primary metabolism could be reduced when *P*. *indica* is present. The strong reduction of these proteins in the exudate preparations might be due to their easier accessibility for fungal proteases. The high number of putative degrading enzymes in the *P*. *indica* secretome supports this hypothesis. However, whether plant proteins involved in the primary metabolism are specifically degraded, is unclear. An interpretation of these results requires more information about the targets of the fungal proteases, which is still poor.

We also found proteins associated with mucilage in the media of the different cultures. Recent evidence points to mucilage function in seed germination, root tip growth and the formation of arbuscular mycorrhiza [93–95]. The importance of mucilage for arbuscular mycorrhiza has been shown for several plant symbioses including the interaction of maize roots with *Gigaspora gigantea* in which secreted plant components induce hyphal branching [96]. The function of mucilage during the interaction of Arabidopsis with microbes, such as *P*. *indica*, has not been studied before even though mucilage was investigated in Arabidopsis seeds [73]. Proteins associated with mucilage were identified in the single culture, in the co-culture or in both treatments (S2 Table). For example, the subtilisin-like protease 1.7 (SBT1.7) was present in both cultures (S1 Table), while its homologue SBT1.8 was only detected in the single culture. These quite specific effects suggest that the mucilage-related proteins have specific functions in the symbiosis and are differentially regulated by the fungus. Barely anything is known about the role of mucilage in controlling hyphae entry into root cells, the regulation of plant genes and proteins involved in their synthesis, although it is obvious that they might be an important barrier for fungal invasion, besides other functions which can be envisioned in symbiotic interactions cf. [93–96].

### Secretome from *P*. *indica*

Even though the genome of *P*. *indica* is sequenced, the function of many proteins is still unknown. In our study, most of the differentially regulated proteins were found in the medium of the co-culture. For one of them (PIIN_04899, a putative indole-3-acetaldehyde dehydrogenase), Hilbert and colleagues (2012) showed an induction of this gene by arabinose, a cell wall component of *A*. *thaliana* [97, 98]. For another protein (PIIN_04899), a homologous protein from the *Pseudomonas syringae* strain DC3000 was described. This protein produces indole acetic acid (IAA) via indole-3-acetaldehyde dehydrogenase (ALDA) that catalyses the formation of IAA from indole-3-acetaldehyde. Disruption of *aldA* and a close homolog, *aldB*, leads to reduced IAA production and virulence of DC3000 on *A*. *thaliana*. McClerklin and colleagues (2017) further showed that the IAA produced by this pathway in DC3000 suppresses salicylic acid-mediated defences in Arabidopsis [99]. *P*. *indica* can produce IAA *in vitro* and its growth promoting effect in Arabidopsis can be imitated by this hormone [79]. In the interaction of *P*. *indica* with barley, IAA is required to establish a symbiotic interaction, but not for the growth promotion effect [97]. Hence, the identified fungal protein might trigger the IAA synthesis and, in turn, confer beneficial traits to Arabidopsis.

### Role of PLAT1, ER-bodies and scopolin in the colonisation of *P*. *indica*

We found PLAT1 in the supernatant of the co-culture. PLAT1 functions as positive regulator of abiotic stress tolerance and promotes plant growth [51]. Furthermore, *PLAT1* expression is regulated by the ABA-dependent transcription factors 1–4 [51] which prompted us to have a closer look at the function of the protein in the interaction. In the beginning of the colonisation the expression of this gene is reduced in colonised roots; in confirmation with this results *plat1* mutants are stronger colonised by the fungus after seven days. After two weeks, no difference to the wild-type can be detected. PLAT domains are found in different membrane- or lipid-associated proteins [52, 100–103] and exhibit similarity to the C2 domain involved in protein–protein and protein-membrane interactions [52, 104]. Therefore, it has been hypothesised that the PLAT domain provides a docking platform for proteins to regulate their catalytic activities and that the proteins *per se* do not have enzymatic activities [51–53]. Hyun and colleagues (2014) further observed a co-localisation of PLAT1 with ER-bodies [51]. Since PYK10, which is found in ER-bodies, is involved in the establishment of symbiosis between *P*. *indica* and *A*. *thaliana* [43], one could speculate that besides PYK10, also PLAT1 might play an important role, by regulating defence processes during early phases of the symbiotic interaction. This is supported by the stronger colonisation of *plat1* mutants seven days after colonisation and by the altered level of scopolin in *plat1* roots. Furthermore, the scopolin mutant *f6’h1* plants are stronger colonised by the fungus. This indicates that *F6’H1* or its product scopolin restricts the entry of *P*. *indica* hyphae into roots cells. In agreement with these results, scopolin inhibits growth of *P*. *indica* and other fungi *in vitro* [39]. Downregulation of the *plat1* mRNA in the infected roots seem to be associated with the observed degradation of the ER-bodies, whereas it is not clear whether the expression of *PLAT1* in the nucleus or the ER-bodies in the cytoplasm, or both are the primary target of the fungus in Arabidopsis roots, and whether both effects are directly connected to each other. The appearance of the PLAT1 protein in the exudate fraction might be caused by the disruption of cells, which occurs when the ER-bodies become gradually smaller due to the activation of defence processes against *P*. *indica*. Ultimately, smaller ER-bodies in colonised roots might restrict the liberation of defence compounds such as scopolin from its precursor scopoletin, allowing a more efficient colonisation of roots by the fungus.

Apparently, the early phase of interaction between *A*. *thaliana* and *P*. *indica* includes an arms-race in which *P*. *indica* tries to restrict the plant defence by reducing the amount and function of ER-bodies. At later time points, when a beneficial interaction starts and the fungus is recognized as a friend, the defence compounds PLAT1 and scopolin appear to be less important. These processes can be triggered by altered phytohormone levels. For example, Camehl and colleagues (2010) showed that ethylene can inhibit the fungus and is required for balancing the interaction between *P*. *indica* and *A*. *thaliana* [104]. Other defence-related phytohormones have been reported to be activated during different phases of the symbiotic interaction. Although they may explain alterations in the expression of genes involved in scopolin/scopoletin biosynthesis after exposure of the roots to *P*. *indica*, the role of PLAT1 and the coumarins in this scenario require more investigation.

## Conclusion

In this study, we analysed the secretion of proteins during the early interaction between *A*. *thaliana* and *P*. *indica*. The identified fungal proteins demonstrate that root colonisation results in an almost complete alteration in the fungal exudation profile. Unfortunately, barely anything is known about the function of the secreted fungal proteins, making it difficult to draw meaningful conclusions.

Several of the identified Arabidopsis proteins are differentially regulated. They function in defence and might be required to restrict entry of the hyphae into the host cells. This includes remorin 3, germin like protein 4, osmotin like protein 34 or pathogenesis-related protein 5. For other defence-related proteins, a clear function has not yet been described. Our analysis demonstrated that multiple peroxidases and proteins with a role in growth processes, such as patellin 1 and 2 as wells lipid transfer protein 8, were influenced by the fungus. Furthermore, proteins related to mucilage formation, such as RD21A, RD21B or aspartic protease 1, respond to the fungus. The quite unique regulation of the individual mucilage-related proteins in response to the fungus suggests that they play an important and so far, little investigated role, and that symbiosis-specific signals manipulate mucilage formation.

The establishment of symbiosis requires a reprograming of fungal and plant proteins. Benefits for the host only become visible after successful root colonisation and the recognition of the microbe as mutualistic symbiont. Several identified proteins in the single and co-culture suggest that this phase has not yet been reached three days after infection, and that our analyses was performed at a time point when the arms-race between the two symbionts is ongoing.

ER-bodies are involved in defence and the degradation of ER-bodies in response to fungal colonisation might play a role in balancing defence and growth responses. The downregulation of *plat1* by the fungus seems to cause a downregulation of the defence compound scopolin and thus allowing a better colonisation. However, other enzymes and proteins with not well-defined functions are present in the ER-bodies and lower PLAT1 and scopolin levels after root colonisation may only be one aspect in the arms-race that involves ER-body functions.

## Methods and material

### Cultivation of *A*. *thaliana* and *P*. *indica*

Cultivation of *A*. *thaliana* and *P*. *indica* was performed as described before [105]. Briefly, *A*. *thaliana* wild type (Col-0) and mutant seeds (SALK_112728c for *plat1*, cs69080 for GFP-labelled ER-bodies and SALK_132418C for *f6’h1*) were surface-sterilised with a solution containing sterile distilled water (dH_2_O), sodium lauroyl sarcosinate and Clorix (64%, 4%, 32%; v/v/v) for eight minutes under constant shaking, followed by six rinses with dH_2_O. Surface-sterile seeds were sown on a 0.75% (w/v) agar plate. *P*. *indica* (P. indica-JE1) was cultured as described on solid Kaefer medium (KM) (1% (w/v) agar). Arabidopsis seeds were obtained from the Nottingham Arabidopsis Stock Centre [106].

#### Cultivation of *A*. *thaliana* with *P*. *indica* for secretome study

Seeds were planted on MS medium (Murashige and Skoog) [107] in Petri dishes closed with micropore band. After vernalisation at 4°C for two days the plates were kept under an 8 h light (60 μmol photons*m^- 2^*s^- 1^)/ 16 h dark period with a constant temperature of 21°C for 12 days. For the experiment ten petri dishes were filled with 20 ml liquid PNM without sugar for each treatment. For the *P*. *indica* single culture and the co-culture, a fungal plaque with 1 cm diameter was added to the plates and cultivated at room temperature for three days, whereas for the single *A*. *thaliana* culture and the mock control (medium without fungus or plant) a KM plaque with the same diameter was added. After three days, four plants were added to the petri dishes of the *A*. *thaliana* single culture and the co-culture. To the *P*. *indica* single culture and the mock control, a plaque of MS media was added instead. The mock treatment was performed to check for contaminations. After three days, the supernatant was collected and used for the solid phase extraction.

#### Co-cultivation of *A*. *thaliana* with *P*. *indica* for expression and growth promotion studies

For the gene expression study, *A*. *thaliana* and *P*. *indica* were cultivated as described before [105]. A plaque with one cm diameter of *P*. *indica* and KM, respectively, was transferred to petri dishes with PNM covered by a sterilised nylon membrane. After seven days, four twelve-day old seedlings were transferred to the fungal plate and the control plate, respectively. For all experiments, WT and mutant plants were grown in parallel. Roots and leaves were harvested and snap frozen in liquid nitrogen prior further analysis.

### Analysis of secreted proteins

#### Solid phase extraction

Supernatant of liquid spent culture medium was filtered (0.22 μm PES (polyethersulfone) Corning bottle top filter Sigma-Aldrich, Munich, Germany) and treated with a protease inhibitor cocktail (1 tablet cOmplete Ultra, Mini, EDTA (ethylenediaminetetraacetic acid)-free, Easypack (Roche, Basel, Switzerland) per 100 ml medium). Trifluoroacetic acid (TFA) was added to gain a final concentration of 0.1% TFA. Chromabond C4 SPE (solid phase extraction) cartridges (Macherey-Nagel, Düren, Germany) were reconstituted with 3 ml acetonitrile (ACN) and 3 ml 0.1% TFA. Each 20 ml spent medium of 10 biological replicates of plant, fungus, co-culture or mock samples were aspirated (vacuum-driven) through the SPE cartridges. Subsequently, the cartridges were washed with 3 ml 5% MeOH (methanol), 0.1% TFA, and the enriched proteins were eluted from the C4 resin with 3 ml 0.1% TFA in ACN/H_2_O (80%/ 20%, v/v). The eluates were freeze-dried in a Christ alpha 2–4 lyophilizer (Christ, Osterode am Harz, Germany). The lyophilised powder was resolubilised in 100 μl denaturation buffer, i.e. 50 mM TEAB (triethylammonium bicarbonate) in 50/50 trifluoroethanol (TFE)/H2O (v/v) by ultrasonification in the water bath for 15 min and heat treatment at 90°C for 10 min. For reduction of oxidised cysteine thiol residues 4 μl 500 mM TCEP (tris(2-carboxyethyl)phosphine) in 100 mM TEAB was added for 1 h incubation at 55°C. Cysteine thiol alkylation was performed by addition of 4 μl 625 mM iodoacetamide in 100 mM TEAB and incubation at room temperature in the dark for 30 min. Proteins were cleaned-up by MeOH/H_2_O/chloroform precipitation using the protocol of Wessel and Flügge (1984) [108] and the precipitate was resolubilised in 100 μl of 100 mM TEAB by 15 min ultrasonic bath treatment. Total protein concentration was determined by the Merck Millipore Direct Detect system (Merck, Darmstadt, Germany) according to the manufacturer’s instructions. Trypsin and LysC protease mix (Promega Cat. # V5072) was added to the samples at a ratio of 1:25 protease mix to protein. Proteins were digested for 18 h at 37°C. Reactions were stopped by addition of 10 μl 10% HCOOH, and the samples were dried in a SpeedVac (Thermo Fisher Scientific, Waltham, MA, USA). Tryptic peptides were resolubilised in 25 μl 0.05% TFA, 2% ACN by 15 min ultrasonic bath treatment. Finally, samples were filtered through 10 kDa PES molecular weight cut-off centrifugal filters (VWR International, Radnor, PA, USA) for 15 min at 16000 g transferred into HPLC (high-performance liquid chromatography) vials and stored at -80°C until LC-MS/MS analysis.

#### LC-MS/MS analysis

LC-MS/MS analysis was carried out on an Ultimate 3000 RSLC Nano system coupled to a QExactive HF mass spectrometer (both Thermo Fisher Scientific). Peptides were enriched online using a nano trap column (Acclaim Pep Map 100, 2 cm x 75 μm, 3 μm, Thermo Fisher) at a flow rate of 5 μl/min. Further peptide separation on an analytical column was performed on an Acclaim Pep Map RSLC nano column (50 cm x 75 μm, 2μm) (Thermo Fisher Scientific). The mobile phase consisted of eluent (A) 0.1% (v/v) formic acid in H_2_O and eluent (B) 0.1% (v/v) formic acid in ACN/H_2_O (90%/ 10%, v/v). Gradient elution was performed as follows: 0 min at 4% B, 5 min at 6% B, 25 min at 8% B, 65 min at 20% B, 80 min at 30% B, 90 min at 50% B, 95–100 min at 96% B, 100.1–120 min at 4% B.

A stainless-steel emitter in the Nanospray Flex Ion Source (Thermo Fisher Scientific) was used to generate positively charged ions at a spray voltage of 2.2 kV. The quadrupole/orbitrap instrument was operated in Full MS / data-dependent MS2 (Top15) mode. Precursor ions were monitored at m/z 300–1500 at a resolution of 120k FWHM using a maximum injection time (ITmax) of 100 ms and an AGC (automatic gain control) target of 1e6. HCD fragmentation at 30% normalised collision energy (NCE) generated MS2 ions, which were scanned at 15k FWHM using a maximum ITmax of 100 ms and an AGC target of 2e5. Dynamic exclusion of precursor ions was set to 30 s. The LC-MS/MS instrument was controlled by Chromeleon 7.2, QExactive HF Tune 2.8 and Xcalibur 4.0 software (Thermo Fisher Scientific).

#### Protein database search

Tandem mass spectra were searched against the UniProt database of *A*. *thaliana* and *P*. *indica* (2017/02/05) using Proteome Discoverer (PD) 2.1 (Thermo Fisher Scientific) and the algorithms of Mascot 2.4 (Matrix Science, London, UK), Sequest HT (integral version of PD 2.1) and MS Amanda 1.0. Two missed cleavages were allowed for trypsin digestion. The precursor mass tolerance was set to 10 ppm and the fragment mass tolerance was set to 0.02 Da. Modifications were defined as dynamic methionine oxidation and static cystheine carbamidomethylation. At least 2 peptides per protein and a strict false discovery rate (FDR) < 1% were required for positive protein hits. The mass spectrometry proteomics data have been deposited to the ProteomeXchange Consortium via the PRIDE [109] partner repository with the dataset identifier PXD009563.

For the GO-Term analysis, all identified proteins were searched against the GO consortium database using AmiGO (http://www.geneontology.org, version 1.8) [55–57]. The frequency (in %) of a specific GO-Term was determined individually for each treatment and biological replicate. The mean frequency for the different treatments was calculated and the 20 most abundant terms were used for further analysis.

The algorithms TargetP 1.1 (http://www.cbs.dtu.dk/services/TargetP/), SignalP 4.1 (http://www.cbs.dtu.dk/services/SignalP/) and Predotar 1.3 (https://urgi.versailles.inra.fr/Tools/Predotar) were used for predicting subcellular localisation [76, 77].

## Additional analysis

### Gene expression studies

The co-culture of *P*. *indica* and *A*. *thaliana* was performed as described before [105]. Roots and leaves were collected for RNA extraction and cDNA (complementary Deoxyribonucleic acid) synthesis followed by RT-qPCR (reverse transcriptase quantitative polymerase chain reaction) as described in [81]. All experiments were repeated in three to four biological and three technical replicates. Primers are given in S3 Table. The efficiency of expression was manually determined by LinRegPCR 2014 [110] followed by the ΔΔ Ct (threshold cyclce) method [111]. Expression levels of genes were normalised by glyceraldehyde 3-phosphate dehydrogenase (*GAPDH*), whereas the relative normalised expression depends on the respective control as stated in the figures.

### Microscopy

Three, seven and 14 days after infection of *A*. *thaliana* with GFP-labelled *P*. *indica* (gift from Prof. P. Schäfer, University Warwick) root colonisation was imaged using an LSM (laser scanning microscope) 880 (Zeiss Microscopy GmbH, Jena, Germany), with the 488 nm laser line of an argon multiline laser. Images were taken with a 40x objective (Plan-Apochromat 40x/0.8). Pictures for quantification of ER-bodies were taken with an Axiolmager M2 microscope (Zeiss). Digital images were processed using ZEN (ZEISS Efficient Navigation) software.

### Extraction of scopolin/ scopoletin from plant material for HPLC analysis

The procedure is based on Döll [112]. For 100 mg frozen plant material, 400 μl methanol and 2mM trans-cinnamic acid (Sigma-Aldrich) per sample as internal standard was added and homogenized for 60 seconds at full speed by an electric homogenizer (RZR 2102 Control (Heidolph, Schwabach, Germany). The samples were transferred to a centrifuge tube and centrifuged at 15.000 rpm and 4°C for 10 min. The supernatant was transferred to a new tube and the pellet resolved in 400 μl methanol. After a second centrifugation step, the supernatants of identical samples were mixed and centrifuged again for 10 min. Finally, the samples were filtered through a 20 μm filter into a glass vial.

### HPLC measurement

For the measurement a JASCO HPLC 900 system (JASCO Germany GmbH, Gross-Umstadt, Germany) was used. It was equipped with a DG-2080-53 degaser, a LG-980-02 solvent mixer, a PU-980 vacuum pump, an AS-950-10 autosampler and a MD-910 multiwave length detector. We used C18 column (Kromasil C18 HPLC column 5μm particle size, pore size 100 Å, length 150 mm, inner diameter 4.6 mm, K08670356, Sigma-Aldrich) at 20°C with a flowrate of 0.6 ml/min. The device was coupled via a JASCO LC-Net II/ADC interface with the computer controlled by ChromPass/Galaxie 1.10.0.5590 from JASCO. Purging was performed for 1 min at a flow rate of 5 ml/min with 0.1% (v/v) formic acid in acetonitrile. This was followed by degassing with 0.1% (v/v) formic acid in water for 1 min at the same flowrate. The column was washed with 0.1% (v/v) formic acid in acetonitrile for ten minutes. For equilibration 2% (v/v) acetonitrile + 0.1% (v/v) formic acid in 98% (v/v) water + 0.1% formic acid (v/v) was used until constant pressure was reached. 10 μl of sample were injected and eluted over 27 min. In the beginning the acetonitrile content in the mobile phase was 2% (v/v) increasing to 100% over 15 min kept at 100% for 5 min, decreased to 2% over 1 min and kept for 6 min at 2%. As an authentic standard 10 μl of 0.1 mM scopolin and 10 μl of 0.625 mM scopoletin (Phytolab, Vestenbergsgreuth, Germany) were used. The amount of scopolin and scopoletin in the sample was determined by the peak area normalised with the peak for *trans*-cinnamic acid (Sigma-Aldrich).

### Growth inhibition experiment of *P*. *indica* by scopoletin and scopolin

The procedure was performed as described by [113]. Scopolin and scopoletin was purchased from Sigma-Aldrich and diluted in methanol. Scopolin, scopoletin and methanol (for the control) were added to six-well plates. After evaporation of the solvent, the wells were filled with 1.5 ml liquid KM medium. 4.5x10^5^ fungal spores were added per well. After 11 days of growth, *P*. *indica* was harvested and dried (60°C, overnight) to determine the dry weight for calculation of the inhibition of fungal growth in %. Bars represent standard deviation. The experiment was repeated in three biological replicates.

### Statistics and data deposition

All statistic was performed by Sigma Plot 13.0.083 (Systat Software, Erkrath, Germany). Details are given in the figure legends.

The MS proteomics data in this paper have been deposited via ProteomeXchange with identifier PXD009563.

## Supporting information

S1 Table List of proteins identified in the secretomes from single and co-cultures. Selected proteins were identified by at least two unique peptides and a false discovery rate of <1%. The frequency of occurrence is given as Low (1 out of 3), Medium (2 out of 3) or High (3 out of 3 replicates) based on the frequency of detection in all replicates.
(XLSX)

S2 Table List of proteins enriched across all three secretomes. Identified proteins were considered differentially enriched, when they were found in at least two replicates in one treatment and not in the other treatment. Furthermore, proteins that were found with a high frequency in one treatment and with a low frequency in the other treatment, were considered differentially secreted as well. Information about the function was obtained using the BLAST algorithm for homology searches against the UniProt database [54, 114]. The location was predicted using TargetP, SignalP and Predotar algorithms.
(XLSX)

S3 Table List of primers used in this study. (XLSX)

S1 Fig Colonisation of *A*. *thaliana* by *P*. *indica* in liquid media at 3 dpi. Arabidopsis roots in liquid (A) and solid media (B) were imaged using fluorescence microscopy. In both cultures, only little fungus was found in and around the root. The first pictures show the autofluorescence of the root and GFP fluorescence of *P*. *indica*. The second pictures show the bright field image. The last pictures show the overlay of all images for each row. Liquid cultivation method from the secretome measurement was compared with the standard solid cultivation method.
(TIF)

S2 Fig Overview of secretomes and Expression analyses of *plat* genes. (A) Venn diagram of *A*. *thaliana* proteins found in three replicates in the co-culture (generated with http://bioinformatics.psb.ugent.be/webtools/Venn/). (B) Profile for *plat2* expression in WT roots and leaves. (C) Profile for *plat3* expression in WT roots and leaves. For *plat3*, CT values were extremely low around 35–40 whereas for all other genes CT values were between 25 and 35. (D) Primer used in this study for RT-qPCR.
Dashed line represents the expression in the untreated WT plants. Error bars represent standard deviation calculated from four biological replicates. Different letters represent significant differences between treatments at p<0.05 (Two-Way ANOVA, followed by a Bonferroni correction).
(TIF)

S3 Fig Expression analyses of genes associated with the scopolin pathway. Expression of *PYK10* and other key genes of the scopolin pathway in roots of WT and *F6’H1* at 7 dpi (A) and 14 dpi (B). Error bars represent standard deviation calculated from four biological replicates. The asterisks shows the significant difference, as described in Fig 3A.
(TIF)

S4 Fig Microscopy of ER-Bodies at 3 dpi in liquid media and in solid media. Fluorescence microscopy of ER-bodies in roots without and with *P*. *indica*. Liquid cultivation method from the secretome measurement was compared with the standard solid cultivation method. Mock treatment in liquid culture (A) and in solid culture (B) and colonised roots at 3 dpi in liquid culture (C) and in solid culture (D) are shown. The first pictures show the autofluorescence of the root ER-Bodies. The second pictures show the bright field image. The last pictures show the overlay of all images for each row. Liquid cultivation method from the secretome measurement was compared with the standard solid cultivation method.
(TIF)

S5 Fig Measurement of ER-body size at 3 dpi in liquid media and in solid media. Comparison of the ER-body length in Arabidopsis root cells without and with *P*. *indica* at 3 dpi. Liquid cultivation method from the secretome measurement was compared with the standard solid cultivation method. Error bars represents standard deviation. Different letters represent significant differences between treatments at p<0.05 (Two-Way ANOVA, followed by a Bonferroni correction). Per time point and treatment four roots were used and the size of 20–100 ER-Bodies per root was measured.
(TIF)
