# Supplementary figures and images for: Arabidopsis thaliana responds to colonisation of Piriformospora indica by secretion of symbiosis-specific proteins
Source: PLoS One. 2018 Dec 27;13(12):e0209658. doi: 10.1371/journal.pone.0209658 (PMC6307754; doi:10.1371/journal.pone.0209658)

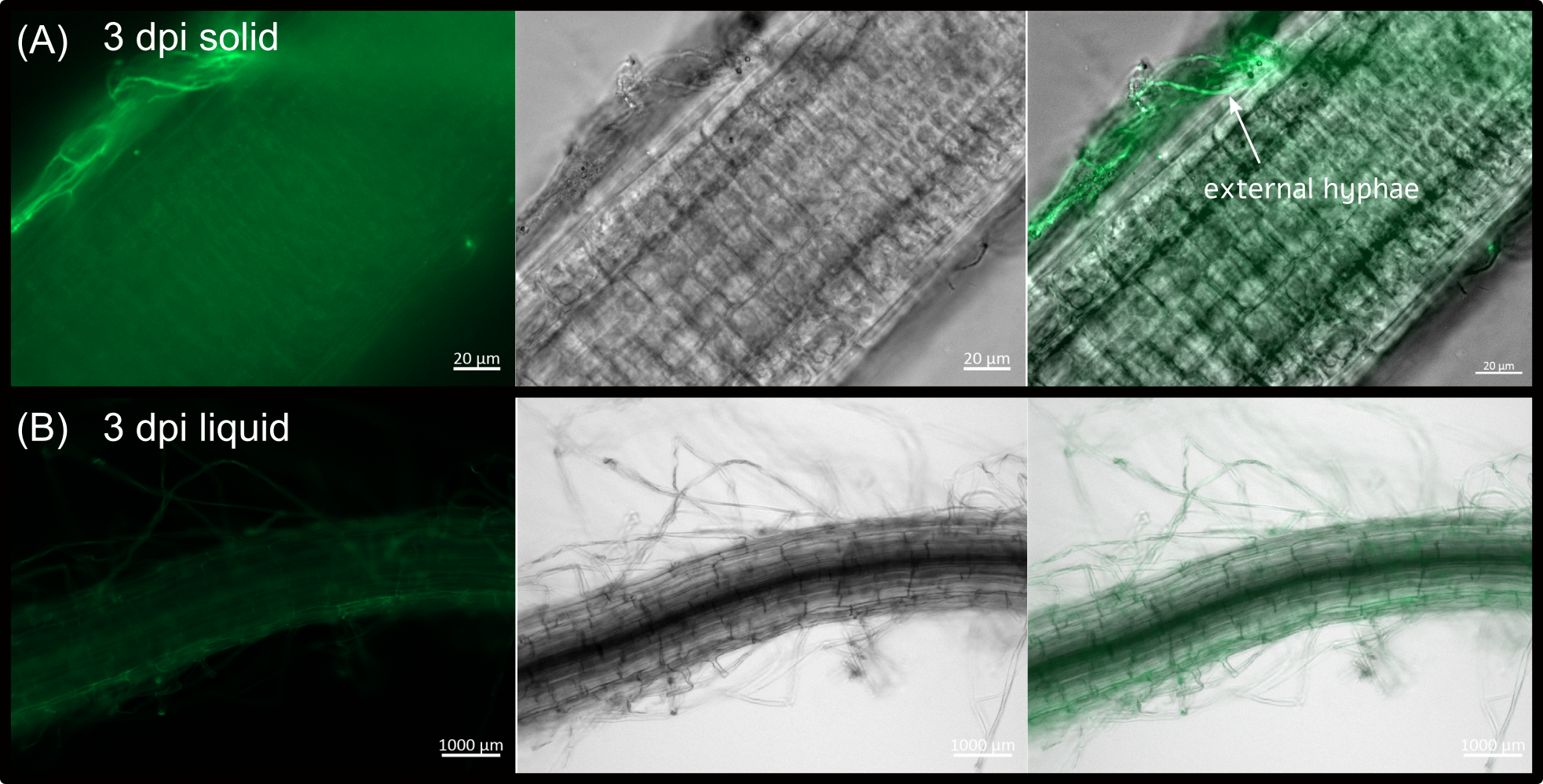

Supplement: S1 Fig — Arabidopsis roots in liquid (A) and solid media (B) were imaged using fluorescence microscopy. In both cultures, only little fungus was found in and around the root. The first pictures show the autofluorescence of the root and GFP fluorescence of P. indica. The second pictures show the bright field image. The last pictures show the overlay of all images for each row. Liquid cultivation method from the secretome measurement was compared with the standard solid cultivation method. (TIF) [file pone.0209658.s004.tif]

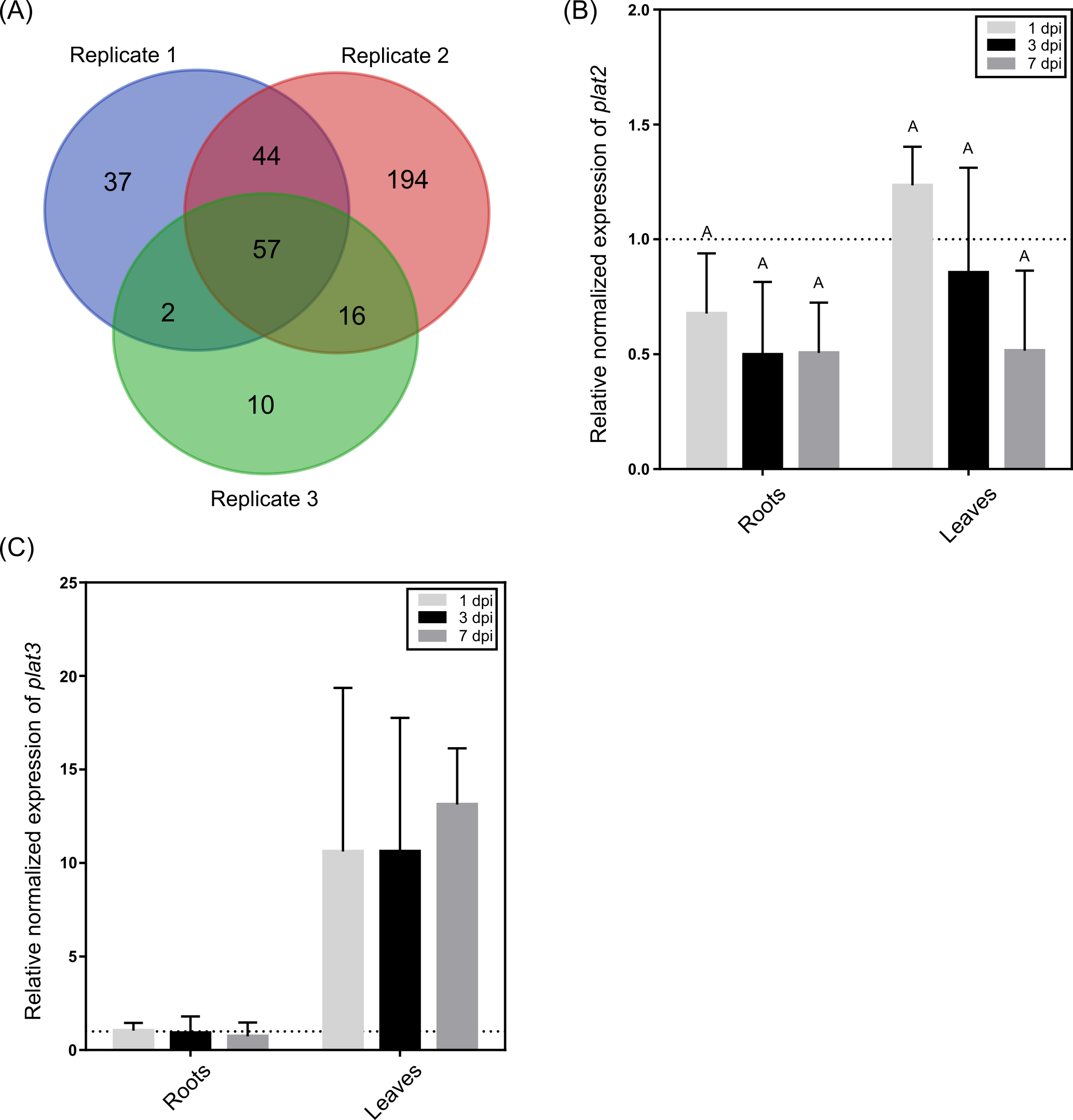

Supplement: S2 Fig — (A) Venn diagram of A. thaliana proteins found in three replicates in the co-culture (generated with http://bioinformatics.psb.ugent.be/webtools/Venn/). (B) Profile for plat2 expression in WT roots and leaves. (C) Profile for plat3 expression in WT roots and leaves. For plat3, CT values were extremely low around 35–40 whereas for all other genes CT values were between 25 and 35. (D) Primer used in this study for RT-qPCR. Dashed line represents the expression in the untreated WT plants. Error bars represent standard deviation calculated from four biological replicates. Different letters represent significant differences between treatments at p<0.05 (Two-Way ANOVA, followed by a Bonferroni correction). (TIF) [file pone.0209658.s005.tif]

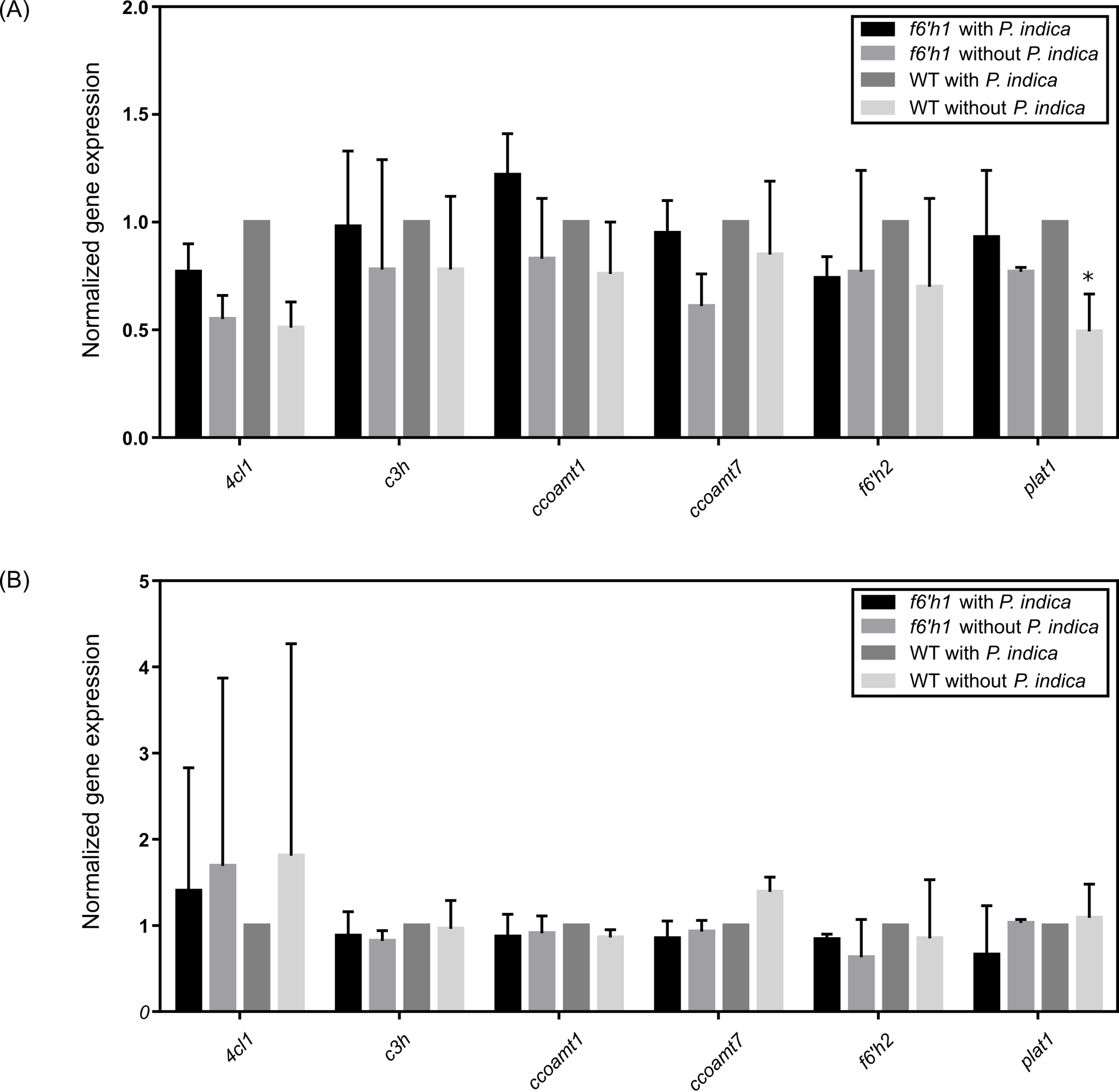

Supplement: S3 Fig — Expression of PYK10 and other key genes of the scopolin pathway in roots of WT and F6’H1 at 7 dpi (A) and 14 dpi (B). Error bars represent standard deviation calculated from four biological replicates. The asterisks shows the significant difference, as described in Fig 3A. (TIF) [file pone.0209658.s006.tif]

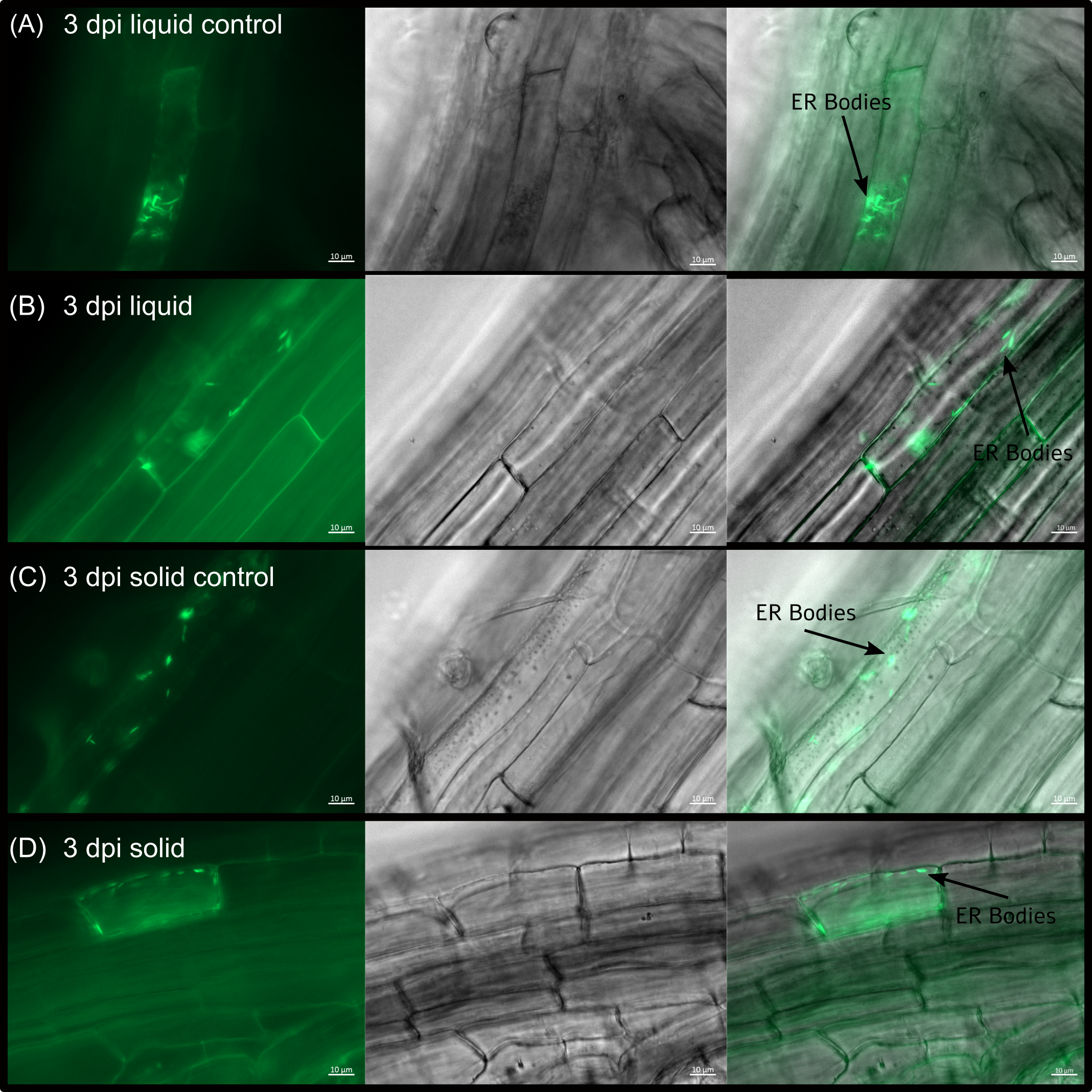

Supplement: S4 Fig — Fluorescence microscopy of ER-bodies in roots without and with P. indica. Liquid cultivation method from the secretome measurement was compared with the standard solid cultivation method. Mock treatment in liquid culture (A) and in solid culture (B) and colonised roots at 3 dpi in liquid culture (C) and in solid culture (D) are shown. The first pictures show the autofluorescence of the root ER-Bodies. The second pictures show the bright field image. The last pictures show the overlay of all images for each row. Liquid cultivation method from the secretome measurement was compared with the standard solid cultivation method. (TIF) [file pone.0209658.s007.tif]

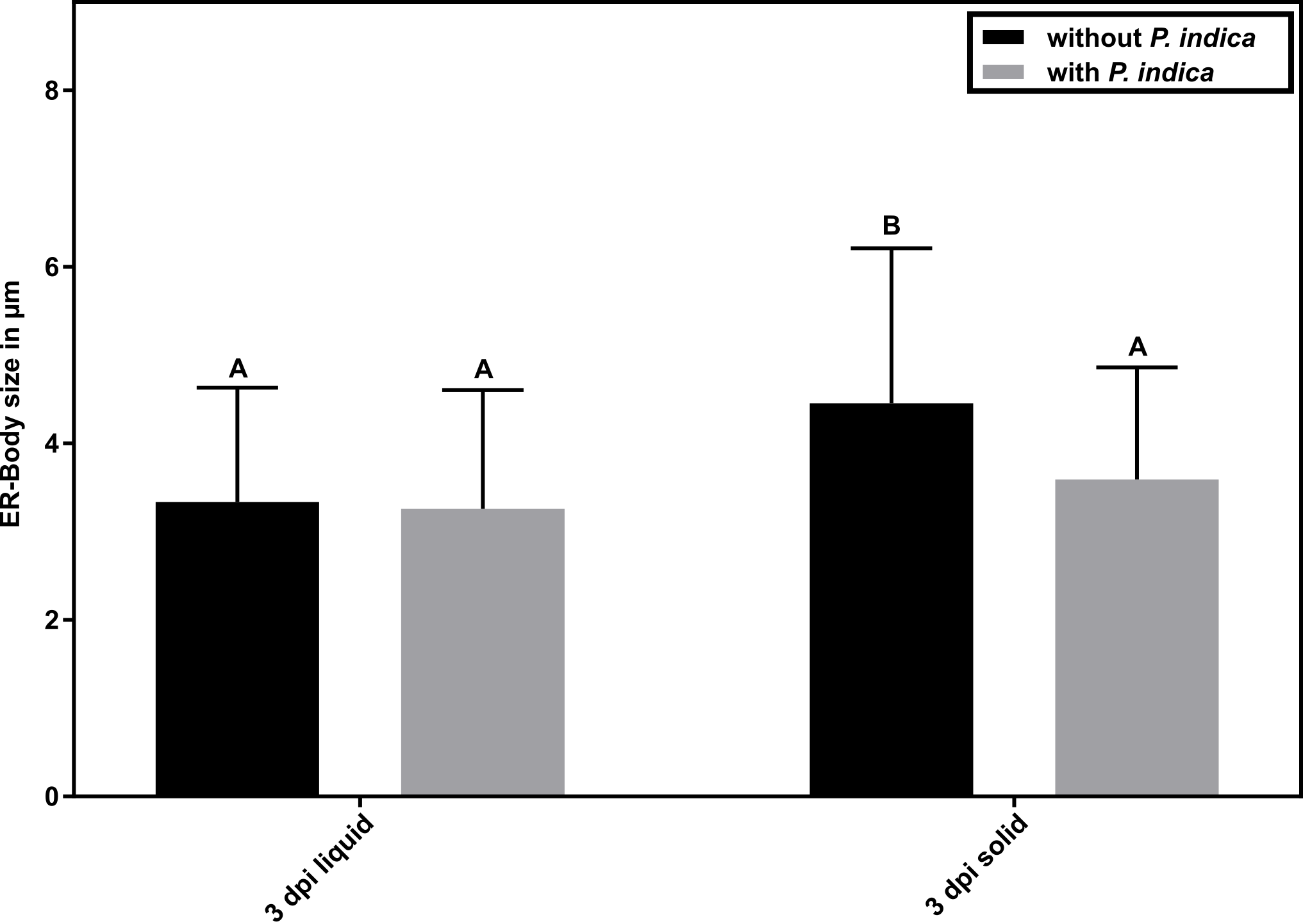

Supplement: S5 Fig — Comparison of the ER-body length in Arabidopsis root cells without and with P. indica at 3 dpi. Liquid cultivation method from the secretome measurement was compared with the standard solid cultivation method. Error bars represents standard deviation. Different letters represent significant differences between treatments at p<0.05 (Two-Way ANOVA, followed by a Bonferroni correction). Per time point and treatment four roots were used and the size of 20–100 ER-Bodies per root was measured. (TIF) [file pone.0209658.s008.tif]
